# Murine colon proteome and characterization of the protein pathways

**DOI:** 10.1186/1756-0381-5-11

**Published:** 2012-08-28

**Authors:** Sameh Magdeldin, Yutaka Yoshida, Huiping Li, Yoshitaka Maeda, Munesuke Yokoyama, Shymaa Enany, Ying Zhang, Bo Xu, Hidehiko Fujinaka, Eishin Yaoita, Sei Sasaki, Tadashi Yamamoto

**Affiliations:** 1 Department of Structural Pathology, Institute of Nephrology, Graduate School of Medical and Dental Sciences, Niigata University, Niigata, Japan; 2 Department of Physiology, Faculty of Veterinary Medicine, Suez Canal University, Suez Canal, Egypt; 3 Department of Intensive Care Unit, West-China Hospital, Sichuan University, Sichuan, China; 4 Animal resource branch, Center of Bio-based researches, Brain research Institute, Niigata University, Niigata, Japan; 5 Department of Microbiology and Immunology, Faculty of Pharmacy, Suez Canal University, Suez Canal, Egypt; 6 Department of Nephrology, Tokyo Medical and Dental University, Tokyo, Japan

**Keywords:** Colon, Proteome, Mass spectrometry, HPLC

## Abstract

**Background:**

Most of the current proteomic researches focus on proteome alteration due to pathological disorders (*i.e.*: colorectal cancer) rather than normal healthy state when mentioning colon. As a result, there are lacks of information regarding normal whole tissue- colon proteome.

**Results:**

We report here a detailed murine (mouse) whole tissue- colon protein reference dataset composed of 1237 confident protein (FDR < 2) with comprehensive insight on its peptide properties, cellular and subcellular localization, functional network GO annotation analysis, and its relative abundances. The presented dataset includes wide spectra of p*I* and Mw ranged from 3–12 and 4–600 KDa, respectively. Gravy index scoring predicted 19.5% membranous and 80.5% globularly located proteins. GO hierarchies and functional network analysis illustrated proteins function together with their relevance and implication of several candidates in malignancy such as Mitogen- activated protein kinase (Mapk8, 9) in colorectal cancer, Fibroblast growth factor receptor (Fgfr 2), Glutathione S-transferase (Gstp1) in prostate cancer, and Cell division control protein (Cdc42), Ras-related protein (Rac1,2) in pancreatic cancer. Protein abundances calculated with 3 different algorithms (NSAF, PAF and emPAI) provide a relative quantification under normal condition as guidance.

**Conclusions:**

This highly confidence colon proteome catalogue will not only serve as a useful reference for further experiments characterizing differentially expressed proteins induced from diseased conditions, but also will aid in better understanding the ontology and functional absorptive mechanism of the colon as well.

## Background

The essential role of colon in normal physiology includes absorption of vitamins, salts, nutrients and water which is summarized as the final stage digestive process [1,2]. Together with its ability to process indigestible fibers (in certain species) [3], it hosts a wide range of useful microbiota which live in a symbiotic relationship providing a proper absorption and wastes elimination [4]. Recruitment of proteomics in colon studies has been an interest for many researchers especially gastroenterologists [5]. However, overwhelming majorities of these reports were focusing on proteome of disordered colon tissues rather than its normal state. Owing to the *Pubmed-Medline* search result with the keywords “*Colon*” and “*Proteomics*” on June 2012, 300 literatures investigated diseased-state colon proteome (mainly colorectal cancer), while less than 5 partially reported healthy one. To this end, the availability of comprehensive whole tissue- colon proteome remains a critical demand. In a former report by Gourley *et al…,* the authors compared colonic gastrointestinal mucosa by 2 different approaches; 2D and 2DLC. Integration of both analyses resulted in identification of 568 proteins [5]. Another 2DE study examining colonic crypts identified approximately 800 proteins [6], while other comparative experiments compared healthy intestinal scraping versus cell lines (Caco-2) [7], aged intestinal epithelia [8] or colorectal cancer [9-11]. Given the necessity of disclosing colon proteome in its normal state to overcome currently insufficient global understanding of physiological and pathophysiological colonic tissues profiles, our impetus was to provide a comprehensive whole tissue colon proteome as a mainstay for further colon research analysis.

## Materials and methods

### Animals

Three healthy normal C57BL/6 J male mice (8 weeks old) bred at the animal Care Research Center (Niigata University, Japan) was used in the current study. Mice were housed in individual cages in sterile environment with 12 hour light cycle and *ad lib* access to standard chow and filtered water. Experimental animal were treated in accordance with ethics of animal center committee at school of medical and dental sciences, Niigata University [approval number 2009–32].

### Tissue processing and protein extraction

Mice were sacrificed by decapitation and colon; 3 cm above the rectum were longitudinally cut, and rinsed in ice-cold PBS buffer. Tissues were sliced into small pieces prior to homogenization. For colonic protein extraction, 100 mg of wet tissue were homogenized in home- made lysis solution consists of (9.8 M urea, 2% nonidet, 0.2% Ampholite; pH 3–10, 12 μl/ml Destreak buffer, 0.5 μg/ml E-64, 0.5 mM PMSF, 40 μg/ml TLCK, 1.0 μg/μl chymostatin, 0.5 mM EDTA, 0.01% bromophenol blue, and 2 μg/μl aprotonin). Samples were homogenized using Polytron PT1200 homogenizer (Kinematica AG, Switzerland) 5–10 bursts with 45 s interval in ice. Homogenized samples were kept in 37 °C for 1 h with occasional vortex, centrifuged at 12,000 rpm for 20 m. Protein assay for the extracts was carried out using Ramagli’s modified method of Bradford (Bio-Rad, Japan) with bovine serum albumin as a standard [12].

### Protein fractionation, tryptic digestion

Samples were acetone precipitated and reconstituted in SDS sample buffer with 2 β mercaptoethanol (final dilution 4%) [13]. Ten μg of colon protein extract from each sample were run on 12.5% SDS-PAGE. Gel was stained with Coomassie Brilliant Blue stain (CBB R-250, Wako, Japan). Each lane was sliced into 14 consecutive slices (2 cm/slice). Samples were reduced with 10 mM dithiothreitol (DTT), alkylated with 55 mM iodoacetamide (IAA), and digested with 6 ng/μl of trypsin overnight [14]. Peptide was extracted with 0.3% formic acid and 5 μl (0.25 μg digested protein) from each sample was loaded onto nano-LC-ESI-IT-TOF-MS/MS (Hitachi NanoFrontier LD., Tokyo, Japan).

### Reversed- phase capillary Lc-Ms/Ms analysis

Digested peptides were purified and concentrated on a trap column; monolith trap C18-50-150 (Merck, Darmstadt, Germany). Peptides were separated using the C18 separation column; monocap for Fast-Flow, 0.05 × 150 mm. The injected peptides were eluted with 7.5-70% gradient with solvent B (H_2_O/ACN = 98/2 in 0.1%HCOOH) for 120 minutes at 200 nl/minute. Nano-LC-ESI-IT-TOF-MS/MS was performed on the top of two ions in each MS scan. Dynamic exclusion and repeat settings ensured each ion was selected only once and excluded in the subsequent parent ion selection. Precursor ions were selected using the following MS to MS/MS switch criteria: ion range m/z 100–1800, charge state 2–5, and former target ion were excluded for 20 ms. Collision ion dissociation (CID) was performed using nitrogen as collision gas. Data were merged using Mascot daemon (V 2.0) [15].

### Ms/Ms data processing and protein identification

Peak lists were generated using NanoFrontier LD data processing software (V 1.0). Product ion data were searched against Mouse International protein index (IPI_mouse; version 3.71, 169347 entries) using a locally stored copy of the Mascot search engine (version 2.2.1, Matrix Science, London, UK) [15]. The following parameters were used for database search: MudPIT scoring, precursor mass tolerance 0.3 Da, product ion mass tolerance 0.3 Da, 2 missed cleavages allowed, fully tryptic peptides only, fixed modification of Carbamoidomethyl (C), variable modifications of glutamine (Gln) to pyroglutamate (pyro-Glu) (N-term Q); glutamate (Glu) to pyroglutamate (pyro-Glu) (N-term E), Oxidation of histidine and tryptophan (HW); Oxidation of methionine (M), mass values of monoisotopic and peptide charge state of 2+ and 3+. Protein was accepted if at least 2 peptides passed identity and homology threshold of Mascot (MOWSE) algorithm [16]considering that if multiple spectra were identified to match precisely the same sequence and charge state of a given peptide, only the spectrum with highest score was retained. The false discovery rate (FDR) against reversed decoy database was below 2%.

### Relative protein abundance and gene ontology (GO) annotation

To estimate protein contents in the analyzed samples, normalized spectral abundance factor (NSAF) for each protein was calculated [17], in which the total number of tandem mass spectra (SpC) matching peptides of a given protein was divided by its protein length (L), then divided by the sum of (SpC/L) for all uniquely identified proteins in each dataset. Protein abundance factor (PAF) was calculated for each protein where PAF of a given protein is expressed as the total number of non redundant spectra normalized to the molecular weight (KDa) of the cognate protein (10^4^) [18]. Moreover, protein weight% was estimated based on algorithms of exponentially modified protein abundance factor (emPAI) [19]. These parameters were used to rank proteins according to their relative abundance. In addition, enrichment, depletion analysis and functional annotation network for GO terms were visualized and statistically evaluated using BiNGO (v2.3) [20] and ClueGO (v1.1) plug-ins [21] integrated in Cytoscape (v 2.6.3) [22]. To access over- and under- represented GO hierarchies, both plug-ins were setup to two sided hypergeometric statistical testing with significance level (P < 0.05). False discovery rate (FDR) correction was calculated using Benjamini and Hochberg multiple testing correction [23]. A customized and updated GO slim file (OBO v1.2; 32150 term) and gene annotation file were downloaded from GO consortium [24] and Kyoto encyclopedia of genes and genomes (KEGG) pathway databases [25], respectively and used in the current analysis. For secondary structure prediction of membrane and globular proteins, Gravy index (average hydrophobicity or hydrophilicity scores) were measured using Kyte-Doolittle and Hopp Woods formula [26].

## Results and discussion

### Reproducibility

**Figure 1:**
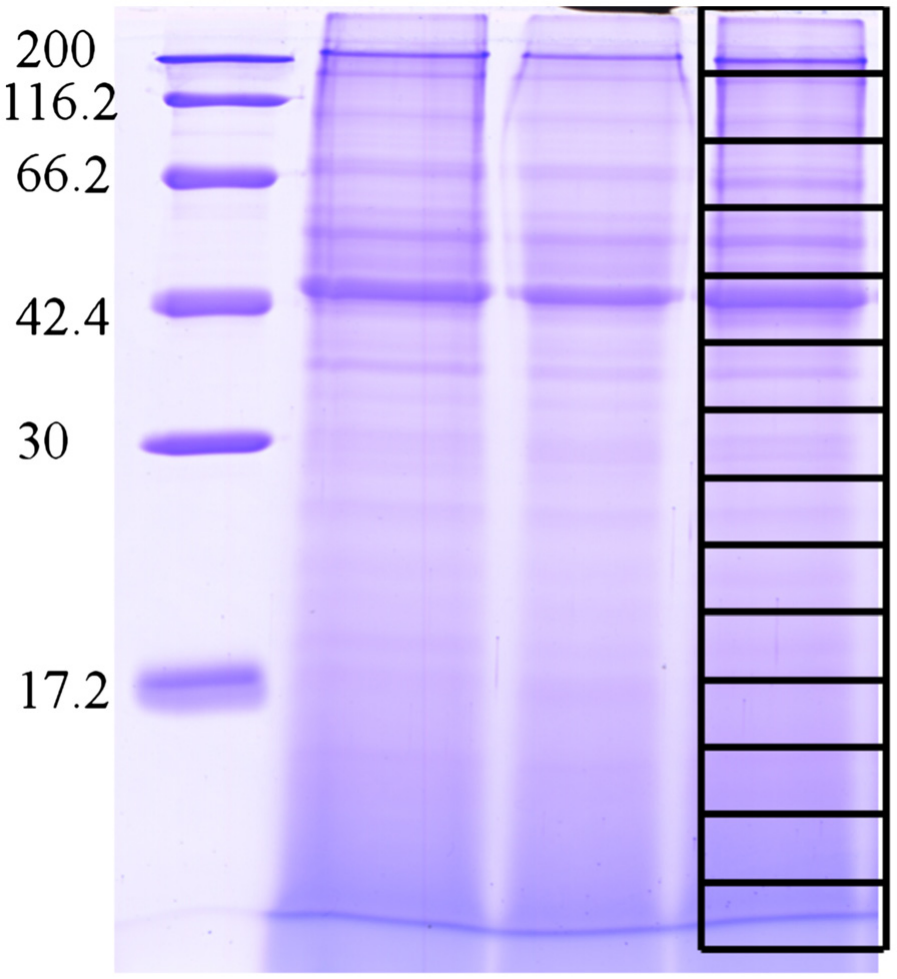
**CBBR-250- stained gel image of protein extract from 3 C57BL/6 J mice colons.** Each lane was sliced into 14 consecutive slices (2 cm/slice) prior to tryptic digestion. High molecular weight marker (BioRad, CA, USA) was loaded in the left lane (KDa).

At first, we examined the reproducibility of our experimental procedures and analysis. Colon tissues were carefully isolated from 3 normal healthy wild type mice. Protein was extracted, run on SDS-PAGE, stained and sliced as shown in Figure 1. Tryptic peptide mixture was eluted, pre-fractionated on C18 separation column and analyzed by Nano-LC-ESI-IT-TOF-MS/MS. Reproducibility of mass analysis was confirmed by matching total ion chromatography (TIC) and peak retention time together with regular monitoring shifts between theoretical and calculated molecular weight precursor ions (Additional file 1) . Moreover, stability of the mass spectrometer was confirmed by performing duplicate check analysis of the same sample which yielded 80-87% similarity of identified proteins (data are not shown). A total of 1237 high confidence proteins with 2 or more peptide matches and FDR < 2 were identified after merging three data sets and removal of redundancy (based on the IPI accession number). Identified protein, including IPI accession number, protein name, peptide matches, theoretical and calculated MW and P*I* are listed in Additional file 2.

### Mouse colon proteome

Using protein data sets generated from 42 slice analysis, we created a proteome catalogue of mouse colon by merging and refining outputs from redundancy and low confidence protein candidates with 1 peptide match. Protein candidate was accepted and considered a confident hit if at least 2 corresponding peptides passed identity and homology threshold of Mascot (MOWSE) algorithm [16]. Over 48.000 Ms/Ms spectra corresponding to 1237 protein candidate were detected and used to configure murine colon proteome (Additional file 2). Ms/Ms spectra of annotated peptides are freely accessible through proteomic data repository, PRIDE, the PRoteomic IDEntification database; http://www.ebi.ac.uk/pride/ under accession number [16857]. Constructed dataset was used for further characterization of mouse colon proteins.

### Characteristics of murine colon proteins

**Figure 2:**
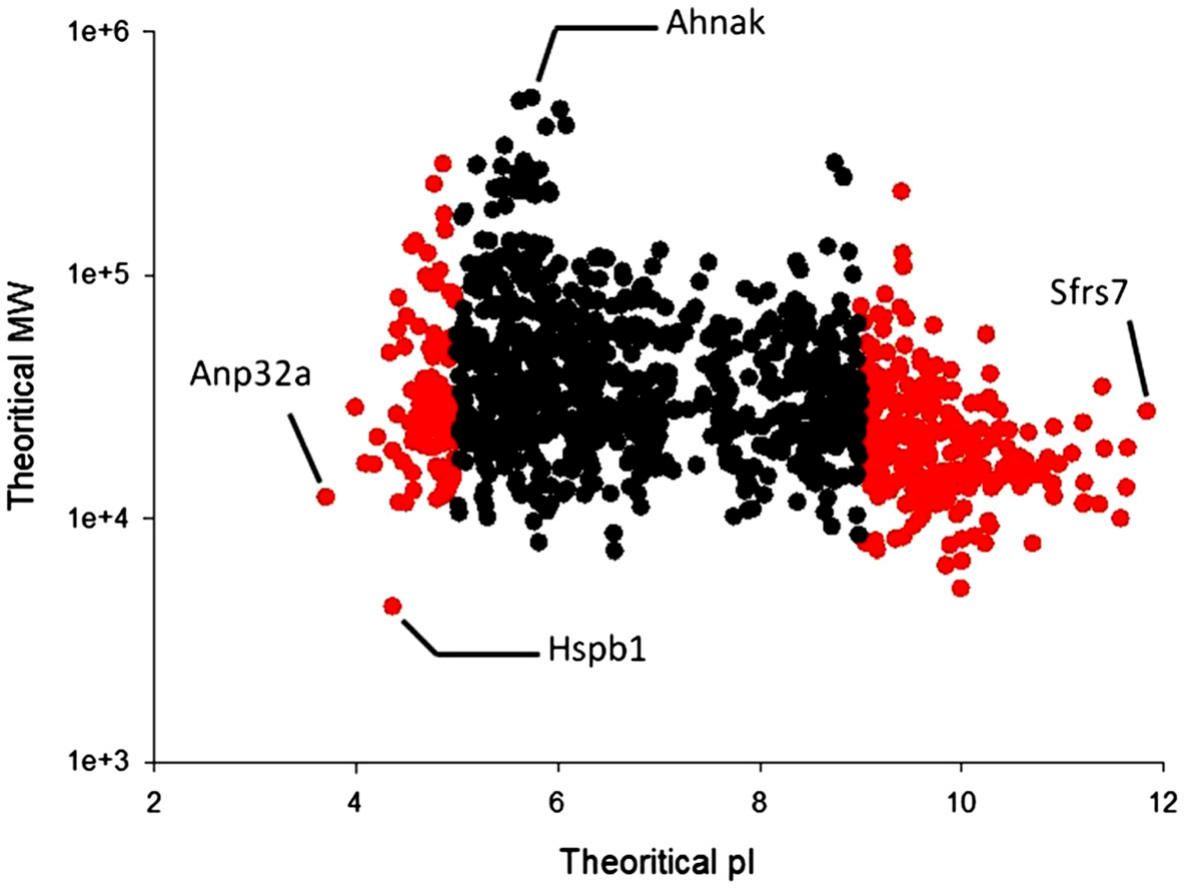
**Theoretical p*I*and MW (Da) of identified proteins (n = 1237).** Black spots represent candidates with p*I* ranged 5 to 9. Red spots represent eccentric proteins with acidic p*I* (3–5) or basic p*I* (9–12). Hspb1; Truncated heat shock protein 1[MW <4 KDa]. Ahnak; neuroblast differentiation- associated protein [MW ~ 600 KDa]. Sfrs7; isoforms 2 of splicing factor arginine/serine-rich 7 [p*I* 11.83]. Anp32a; Acidic leucine- rich nuclear phosphoprotein 32 [p*I* 3.99].

**Figure 3:**
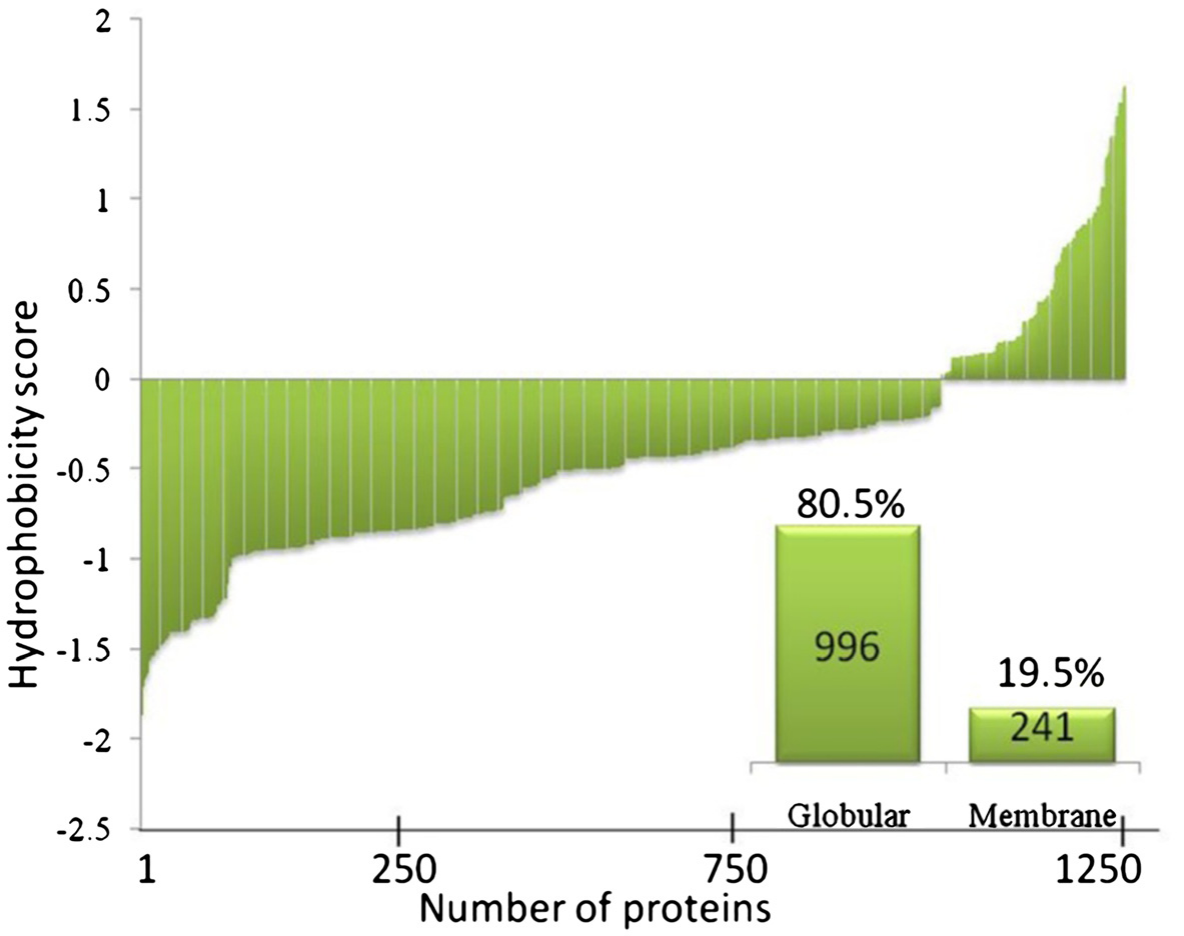
**Gravy index score (average hydrophobicity and hydrophilicity) of colon proteins measured by Kyte-Doolittle and Hopp Woods formula.** Hydrophobicity score (arbitrary unit) below 0 are more likely globular (hydrophilic protein), while scores above 0 are more likely membranous (hydrophobic). Inserted bar panel represents number and percentage of each group in colon proteome.

As illustrated in Figures 2 and 3, we had characterized the identified proteins based on their p*I*, Mw and hydrophobicity. While ~ 68% (n = 843) of proteins were fallen in p*I* ranged from 5–9 and theoretical Mw 10 to 100 KDa, a number of proteins with extreme p*I* or Mw exists in our database; for instance, ~12.5% of identified proteins (n = 155) were reported with acidic p*I* ranged from 3–5 [Anp32a; p*I* 3.99]. Conversely, basic p*I* proteins ranged from 9–12 were representing ~ 19.5% (n = 239) such as Sfrs7 (p*I* 11.83). In addition, we succeeded to resolve proteins with wide Mw ranged from 4 to 600 KDa, for Hspb1 and Ahnak, respectively. These eccentric proteins would rarely be resolved by 2DE. In viewing cellular localization of constructed colon proteome, we further categorized protein candidates into globular or membranous proteins based on amino acids hydrophobicity calculated by Kyte-Doolittle and Hopp Woods formula. Result showed that out of 1237 proteins, 996 representing 80.5% were globular (cytoplasmic) while 241 (19.5%) were membranous (Figure 3). The relative small number of membrane-located proteins might be due to its hydrophobic resistance to be dissolved in the lysis buffer. Further optimization of hydrophobic proteins extraction is required.

### Subcellular localization of identified proteins

**Figure 4:**
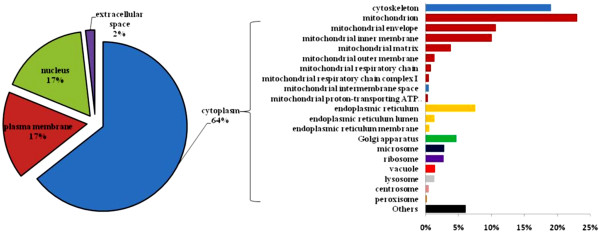
**Subcellular localization of murine colon proteome.** Venn diagram (left panel) shows subcellular distribution. Right panel (bars) represents detailed localization of major cytoplasmic proteins.

We parsed protein dataset into subcellular localization using BiNGO. As shown in Figure 4, around 786 representing 64% of identified proteins were located in cytoplasm. This major compartment was mainly mitochondrial, cytoskeleton, and endoplasmic reticulum proteins (right bar panel). On the other hand, equal numbers of identified proteins were situated in nucleus and plasma membrane (17%; 208 entries/each). A small percentage (2%) of extracellular space proteins was also detected in our dataset.

### GO annotation and functional network analysis

**Figure 5:**
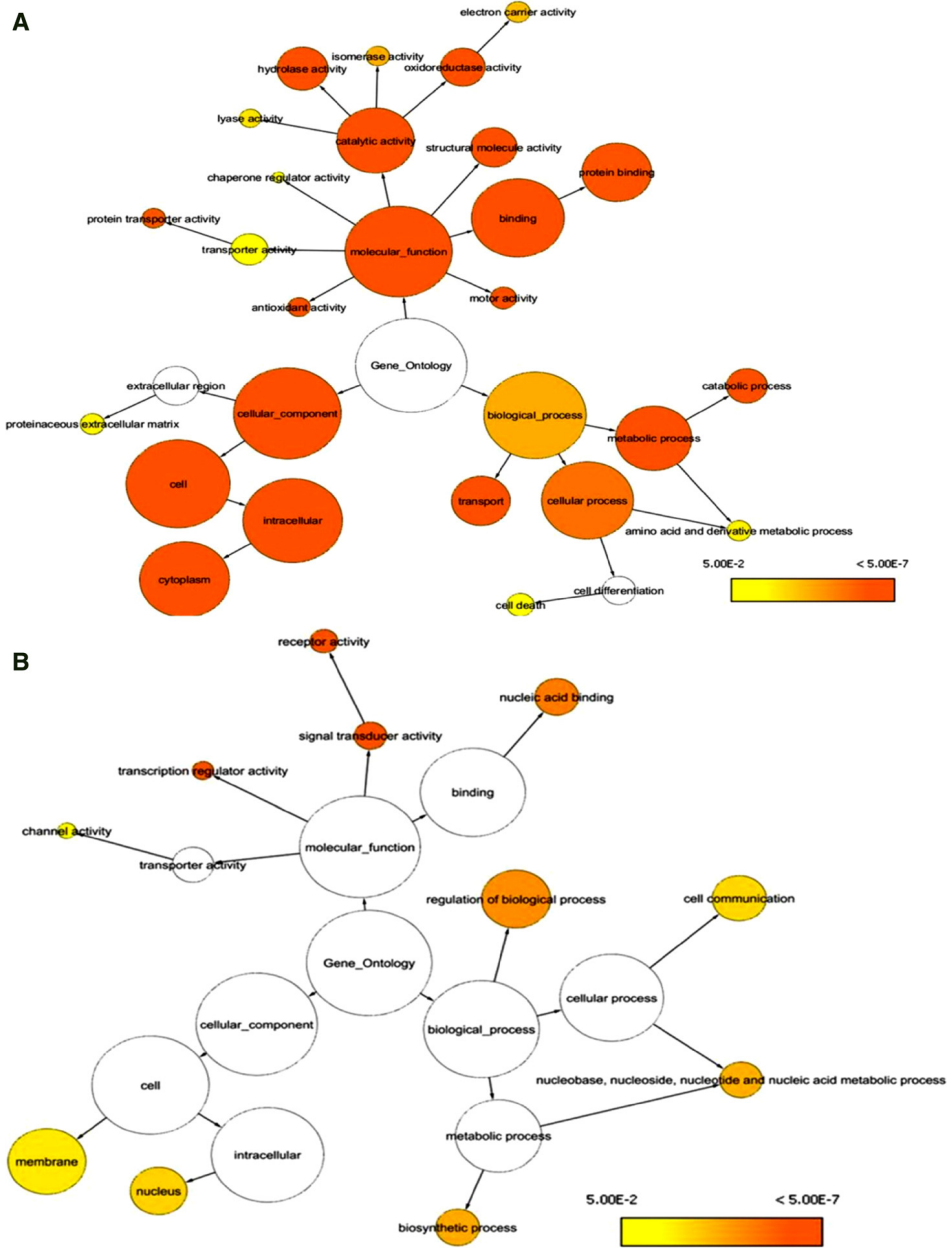
**Gene ontology (GO) biological hierarchy of murine colon proteome.** (**A**) Enrichment analysis (over representative). (**B**) Depletion analysis (under representative). Node size (area) is proportional to the number of GO terms annotated to the node. Node color represents corrected p value ranging from yellow (P < 0.05) to dark orange (5 orders magnitude smaller than yellow). White nodes are non significant.

**Figure 6:**
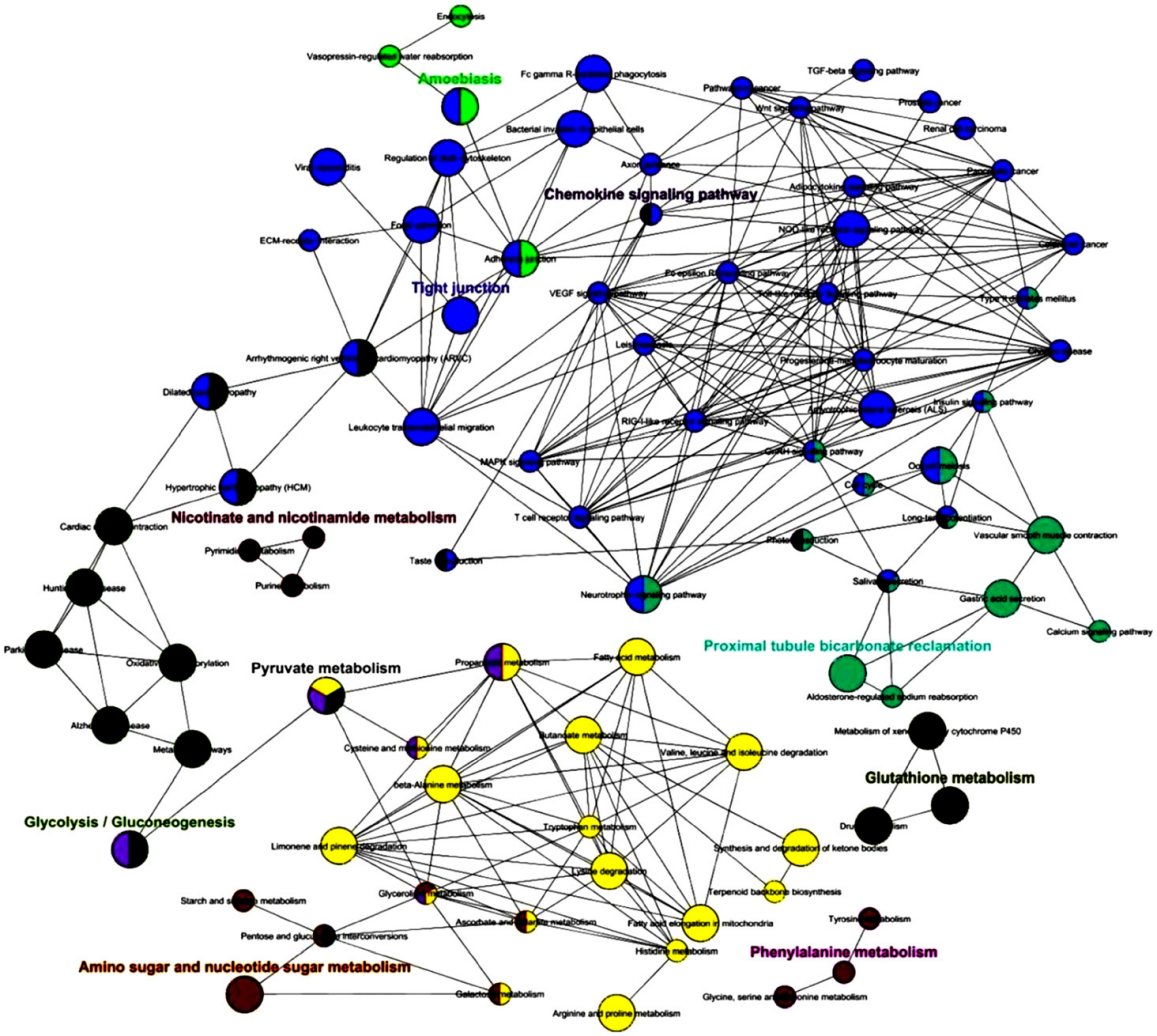
**Functional grouped annotation network reflecting relationship between identified terms based on similarity of their associated genes in mice colon.** Node size reflects statistical significance of the terms. Degree of connectivity between terms (edges) and definition of functional groups (node color) are calculated using kappa statistics. Terms might be included in several groups. Network is laid out using organic layout algorithm supported by cytoscape.

**Figure 7:**
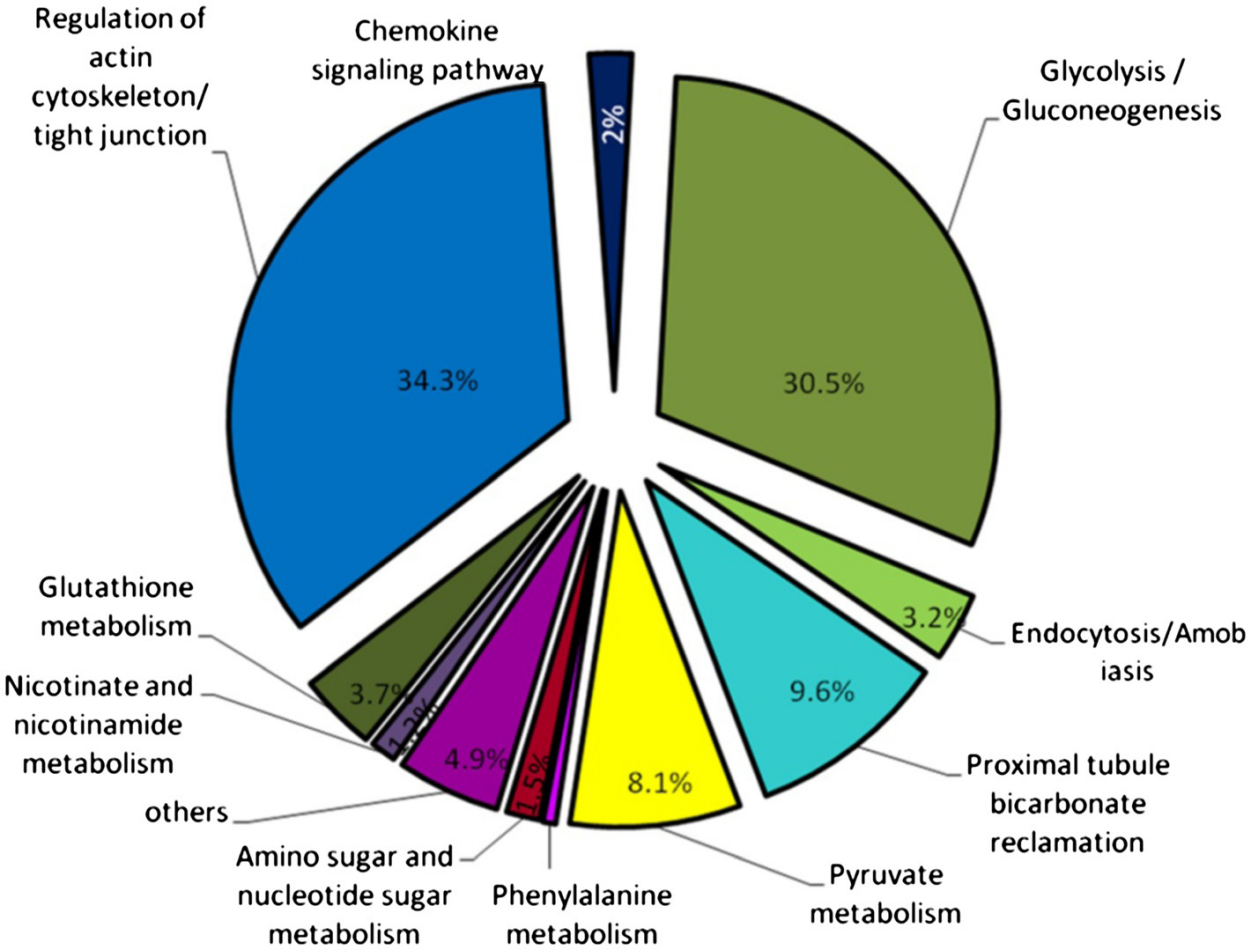
**Overview chart shows the percentage of functional groups in relation to whole murine colon proteome.** The name of the group is given by the group leading term which has the highest significance within the group. Group section size correlates with the number of the terms included in it.

Enrichment and depletion analysis of mouse colon proteome were applied in the current experiment to speculate over and under- represented proteins. As illustrated in Figure 5, enrichment GO annotation showed successful retrieval of wide diversity of proteins (shown by node size and intense color) and reflecting a comprehensive, unbiased Lc-Ms/Ms approach. This observation is coincidental with non significant under representation of the most parent GO categories (colorless nodes in Figure 5B) with an exception of some daughter categories. For instance, membranous and nuclear proteins were shown to be under represented with *P* value less than 0.01 (see also Additional file 3). Once again, this finding supports gravy index result and likelihood is due to the known difficulty to extract membranous proteins because of its hydrophobic nature. Functional analysis and protein family network illustrated in Figure 6 showed the involvement of 1199 identifiers in essential metabolic processes and pathways of the colon tissue (38 identifier were not recognized) and reflect the relationship between the terms based on similarity of their associated genes. As summarized in Figure 7, ten potential groups were recognized and defined with a leading term (to minimize the complex structure of GO tree) based on the highest significance scoring within the group. These enriched groups indicate their relevance in essential metabolic functions and processes in colon tissue. Detailed sub functional term classification can be found in Additional file 1.

### Regulation of actin cytoskeleton/tight junction and related family groups

Representing over one third (563) of identified terms, as illustrated in Figure 6, this family holds 43 GO terms (shown by nodes). Most prominent is actin cytoskeleton which contains 47 identifiers. A wide variety of essential pathways could be recognized in this group. For example; MAPK (proliferation and apoptosis), VEGF (angiogenesis), Toll-like and T-like receptors (Immune barrier) signaling pathways and others, which reflect the active metabolic processes took place in the colonic cells. Moreover, several protein candidates for cancer pathways could be reported. Most notably Mapk8 and 9, Rac1 and 2, Rhoa for colorectal cancer, Cdc42, Fh1, Rac1, Rap1a, Tceb2 for renal cell carcinoma, Cdc42, Mapk8and 9, Pld1, Rac1and 2for pancreatic carcinoma and Fgfr2, Gstp1, Hsp90aa1, Hsp90ab1, Hsp90b1 for prostate cancer.

### Glycolysis and Glyconeogenesis and related family groups

Major 2 ubiquitous processes that involve glucose breakdown (glycolysis) and its generation form non carbohydrate sources (glyconeogenesis) were detected. In colon proteome database, we reported 500 identifiers representing 30.5% of total colon proteome representing Glycolysis pathway. This family holds wide members of enzymes including aldolases A and B, alcohol dehydrogenases family members, enolases (α, ß, and γ), lactate dehydrogenases, and others.

### Proximal tubule bicarbonate reclamation and related family groups

This family includes ATPase, Na+/K + transporters [alpha1-4], glutamate dehydrogenase and malate dehydrogenase 1 (NAD) and representing 9.6% of colon database. These catalytic enzymes are essential for exchanging sodium and potassium ions and providing energy for active transport of various nutrients in the gut. Other ATPase transporters were also identified which are contributors in salivary and gastric acid secretion such as (ATP1b1and ATP4a).

### Pyruvate metabolism and related family groups

An essential group family which has key enzymes in citric acid cycle including dehydrogenases such as pyruvate, lactate, malate dehydrogenase. This group is mainly responsible for cellular respiration and release of energy via NADH. We were able also to recognize a wide variety of enzymes that contribute in amino acid metabolism and participating in cysteine, methionine, valine, leucine and isoleucine, arginine, proline, histidine and tryptophane synthesis and breakdown including LAP3, OAT, GOT2, and ALDH2 (Additional file 3). Several enzymes that share in fatty acid metabolism and elongation were also reported such as acetyl CoA acyltransferese 1 and 2, alcohol dehydrogenase 1 and 2 and others.

### Glutathione metabolism and related family groups

Includes glutathione peroxidase (GPX 1–5) and glutathione S- transferases (GST 1–5) enzymes that protect cells and other enzymes form oxidative damage by catalyzing the reduction of hydrogen peroxide, lipid peroxides and organic hydroperoxides.

### Other families

Representing less than 15% of colon database and including chemokine signaling pathway, nicotinate and nicotinamide metabolism, amino sugar and nucleotide sugar metabolism, phenylalanine metabolism and amobiasis.

### Relative abundance of identified murine colon proteome

**Figure 8:**
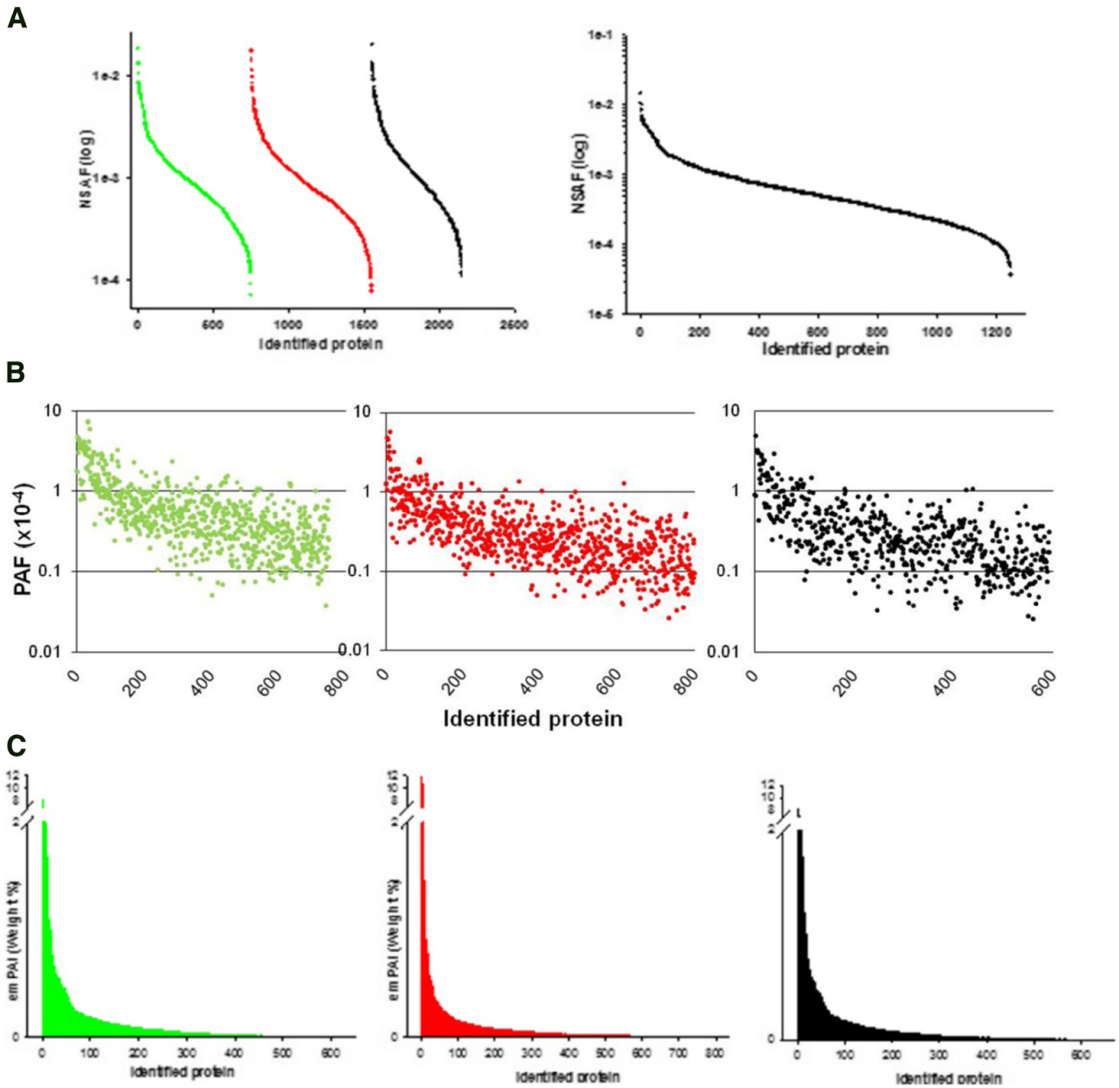
**Relative quantification of murine colon proteins.** (**A**) Normalized spectral abundance factor (NSAF) for each sample (left) and after merging (right). (**B**) Protein abundance factor (PAF). (**C**) Protein weight percentage calculated based on exponentially modified protein abundance factor (emPAI).

In addition to protein identification and annotation, we determined also the relative protein abundance profiling of our murine colon proteome using 3 different approaches; NSAF, PAF, and emPAI owing to its extreme importance in comparative proteomics studies. These algorithms relay on the fact that spectral count (for NSAF and PAF) and peptide rank and score (for emPAI) correlate with relative protein abundance [17-19]. In Figure 8A, individual NSAF in the analyzed samples showed similar pattern. Following construction of the colon proteome catalogue, protein abundance was fallen within the range from 1.2 x 10^-5^ to 1.8 x 10^-2^. This wide dynamic range further attests our unbiased Lc-Ms approach. When calculated using PAF algorithm, most of proteins were ranged from 0.1 x10^-4^ to 1.0 x10^-4^ (Figure 8B). We noticed the existence of several ubiquitous proteins in high abundance (~ 5 folds) such as transgelin, actin, and ß-globin which is recommended to be depleted when investigating low abundance proteins. On the other hand, several low abundance proteins were also identified. For instance, dipeptidyl - peptidase 1 (0.03 x10^-4^) and polymeric immunoglobulin receptor (0.05 x10^-4^). A similar pattern was also noticed when emPAI was used (Figure 8C). To compare between these 3 approaches, we sorted the top 30 abundant peptides (Additional file 4). Obviously, although several top abundant proteins were found to be shared between 3 algorithms, other candidates were different which probably might be due the fact that emPAI relies on peptide score while NSAF and PAF depends on spectral count.

### Comparison of murine colon proteome to gene expression database of mouse colon

**Figure 9:**
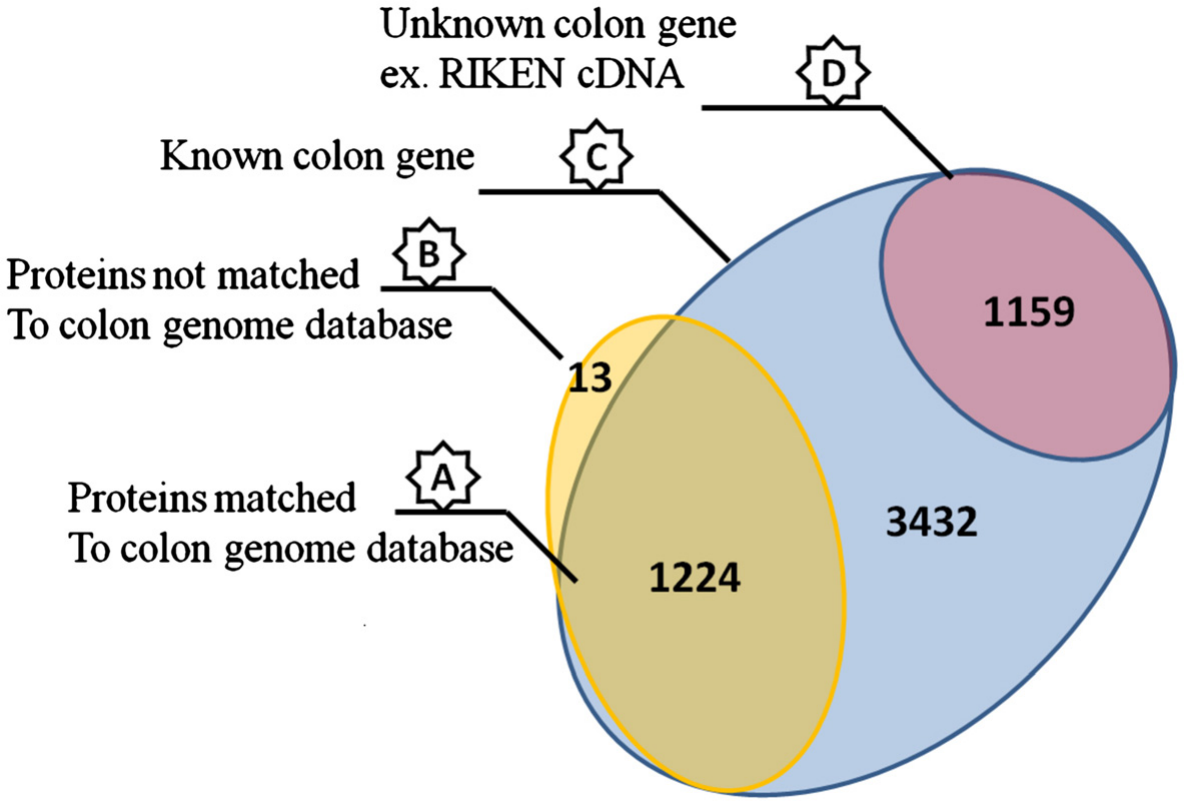
**Venn diagram showing a comparison between our generated proteome catalogue and murine colon gene expression.A**; proteins identified in both proteome and genome database. **B**; proteins identified in proteome database but not in genome database. **C**; known colon genes extracted from genome repository. **D**; unknown colon genes (i,e RIKEN cDNA). **A** and **B** represent colon proteome database (1237), **C** and **D** represent colon genome database.

At last, we compared our generated murine colon proteome with the gene expression dataset for mouse colon in order to confirm the feasibility of this dataset as a standard reference for further colon experimentation. For that purpose, the whole length mouse colon genes were extracted from the reference expression dataset (RefEx) repository. The later was compared versus our generated proteome database (based on its gene ontology). As exemplarily shown in Figure 9, the current murine colon proteome showed an over lapping with around 35.6% compared to the known mouse colon genes. This result can be explained by the selective property and general limitation of mass spectrometry. Moreover, 13 genes were identified in our proteome dataset and have not been recognized in colon genome. These biased genes are possibly a non colon genes contamination.

### Biological insights

Recent advances in mass spectrometric methodologies enabled direct analysis of complex protein mixtures in a shotgun approach for global protein identification and biomarker discovery. Presented data in this article, provides not only a normal comprehensive colon proteome database, but also, various label free quantification methods for researcher’s guidance especially when monitoring cancer- related colon protein expression. Furthermore, a functional network analysis of colon proteome is believed to provide a valuable piece of information for clarifying the relationship between possible predicted biomarkers. For instance, several candidates of gastrointestinal tract carcinoma showed correlated pattern; Mapk8 and Rac1,2 of colorectal cancer, cdc42 of renal cell carcinoma, pld1 in pancreatic cancer and Hsp90b1 and hsp90ab1 in prostate cancer. These data might anticipate in elucidating cell signaling and pathophysiological pathways.

## Conclusion

The present research reports a comprehensive whole-tissue colon proteome catalogue consists of 1237 high confidence candidate protein with FDR < 2, together with its characteristics, relative expression, and functional pathway analysis which depicts unbiased database. Moreover, the colon protein profile shown in this report represents, to the best of our knowledge, a reference point for further comparative studies and better understanding protein expression patterns induced not only in normal physiological status but also in commonly diseased conditions as well.

## Abbreviations

DTT: Dithiothreitol; IAA: Iodoacetamide; LC-ESI-IT-TOF: Liquid chromatography- electospray ionization- time of flight; TFA: Trifluoruacetic acid; SDS-PAGE: Sodium dodecyl sulfate polyacrylamide gel electrophoresis; CID: MOWSE scoring, Molecular weight search scoring; GO: Gene ontology, colloidal ion dissociation; NSAF: Normalized spectral abundance factor; FDR: false discovary rate; PAF: Protein abundance factor; emPAI: exponentially modified protein abundance factor.

## Competing interests

The authors have declared no conflict of interest.

## Authors’ contributions

SM: principally involved in experimental design, sample preparation, analysis and bioinformatic analysis, writing the article. YY carried out the mass spectrometric analysis. HL, YM and MY involved in animal care. SE,BX YZ and HF partially shared in protein alignment and network analysis. EY and SS carried out the genetic analysis and TY shared in experimental design and manuscript revision. All authors read and approved the final manuscript.

## Supplementary Material

Additional file 1 Reproducibility and accuracy of Lc-Ms/Ms analysis for replicate samples. Click here for file

Additional file 2 Murine colon proteome merged database. Click here for file

Additional file 3 Murine colon biological hierarchy showing both enrichment and depletion analysis. Click here for file

Additional file 4 NSAF {bars representing sample replicate runs}. Click here for file
