# Supplementary material for: Murine colon proteome and characterization of the protein pathways
Source: BioData Min. 2012 Aug 28;5:11. doi: 10.1186/1756-0381-5-11 (PMC3497880; doi:10.1186/1756-0381-5-11)
Supplement: Additional file 1 — Reproducibility and accuracy of Lc-Ms/Ms analysis for replicate samples. [file 1756-0381-5-11-S1.pdf]

Supplement 1

<u>Reproducibility and accuracy of Lc-Ms/Ms analysis for replicate samples</u>

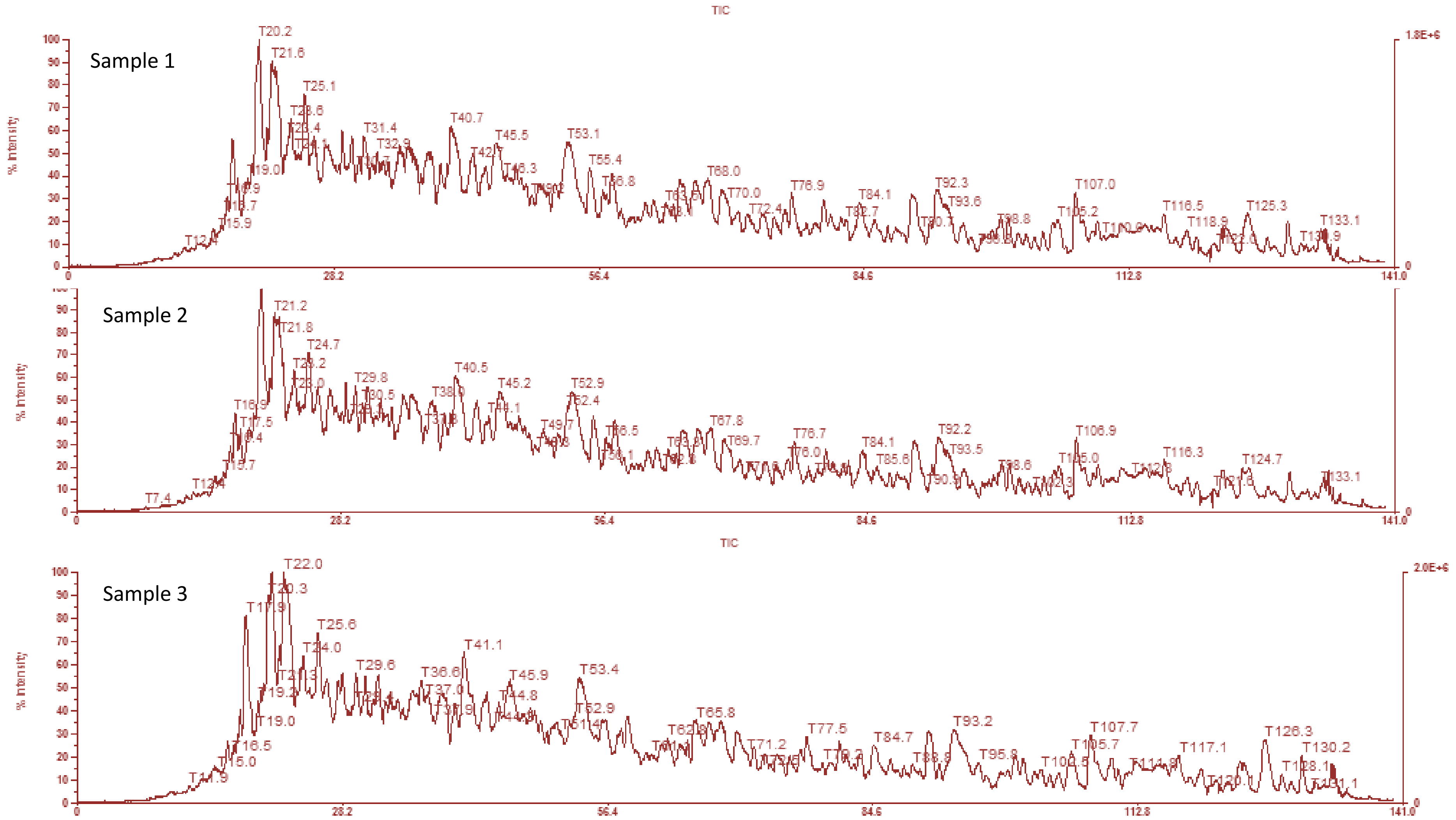

Illustration showing total ion chromatography (TIC) of colon samples used in current experiment. TIC showed a stable and consistent similarity reflecting stability of mass spectrometry. Attention was always monitored to achieve maximum reproducibility and minimizing any variation that might arise from experimental error. Figures were captured using Hitachi NanoFrontier LD data processing software, Tokyo, Japan.

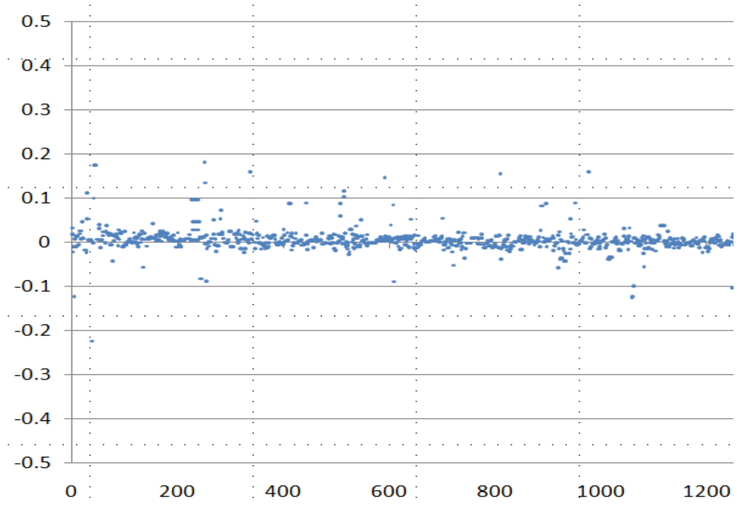

Accuracy of mass spectrometry was confirmed when blotting the delta mass (Δ max); differences between theoretical (Th Mw) and calculated molecular weight (Calc Mw). Shift in most cases were within 0.05 as shown above.

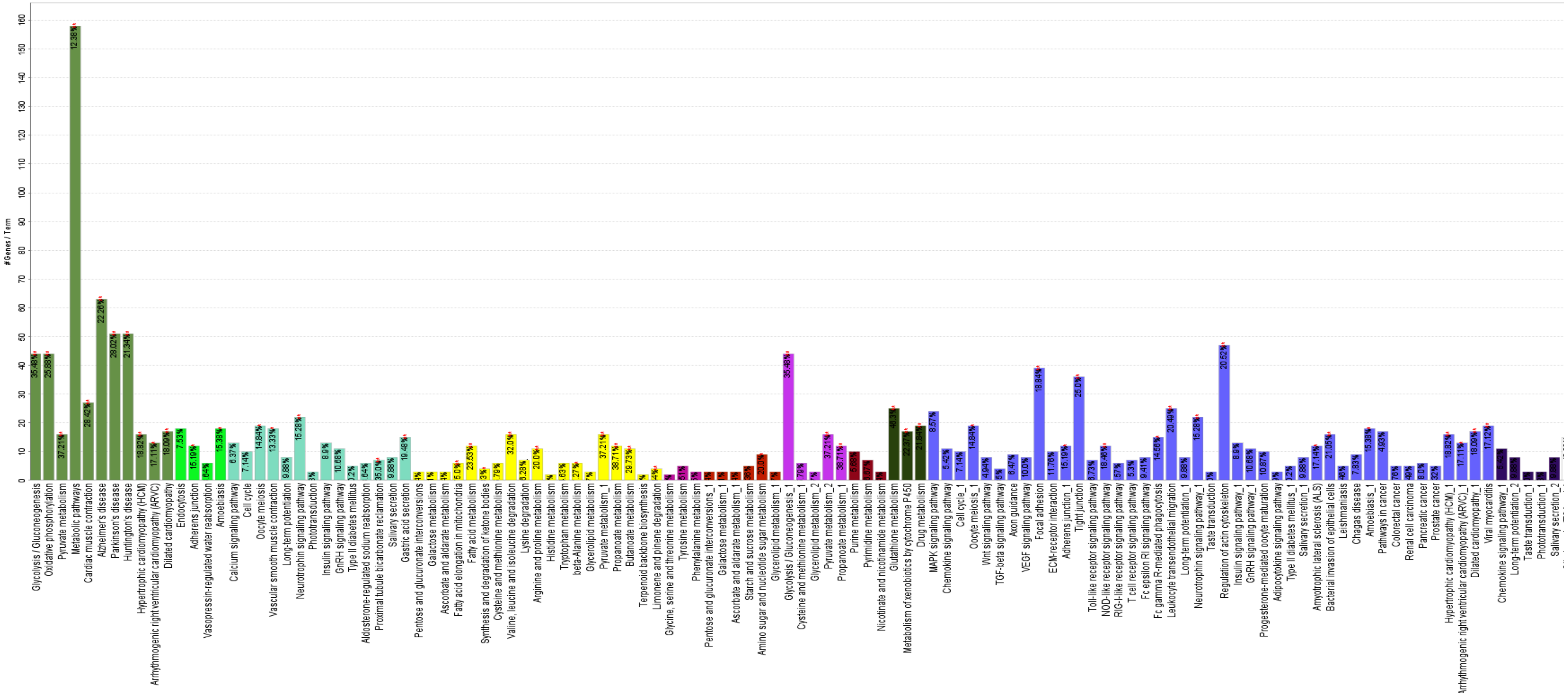

Detailed sub functional classification of annotated murine colon genes, corresponding to murine colon protein proteome. Colors represent 10 major groups. Labels displayed on the bars are percentage of found genes compared to all genes associated with the term. Term significance information is included within the bar graph vertical axis represent number of genes/term.
