# Supplementary figures and images for: Murine colon proteome and characterization of the protein pathways
Source: BioData Min. 2012 Aug 28;5:11. doi: 10.1186/1756-0381-5-11 (PMC3497880; doi:10.1186/1756-0381-5-11)

**NSAF {bars representing sample replicate runs}**

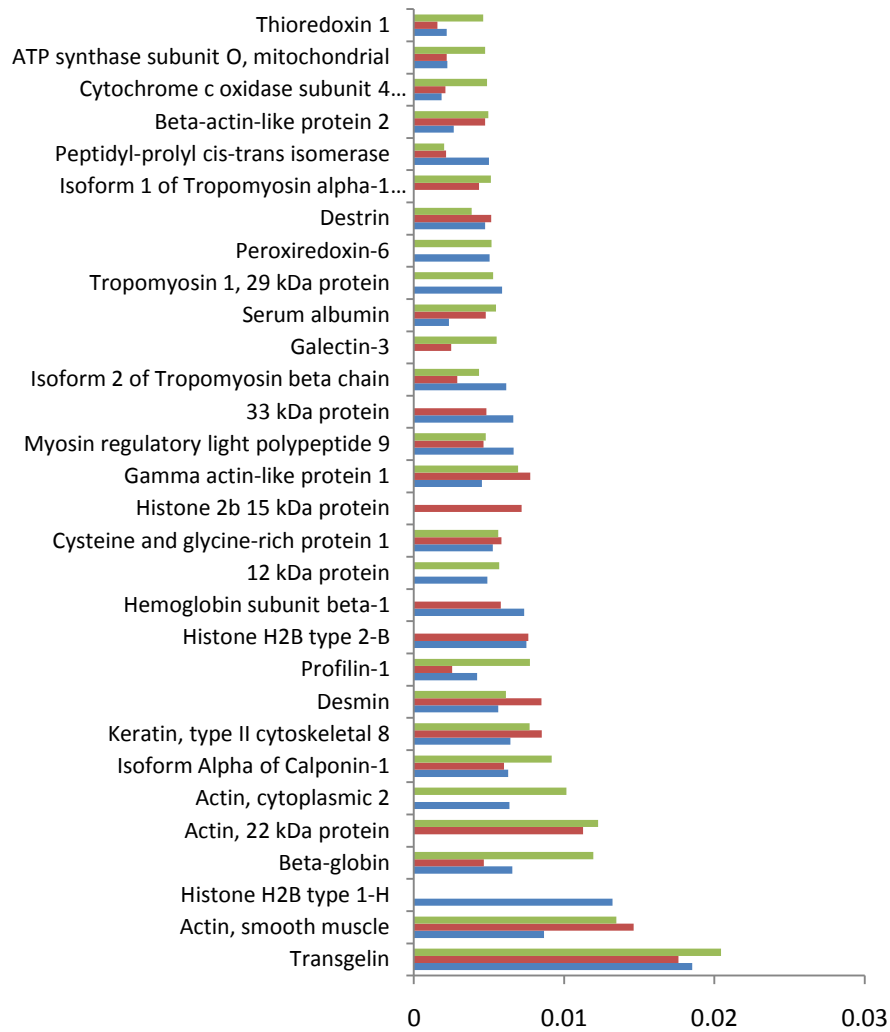

**PAF**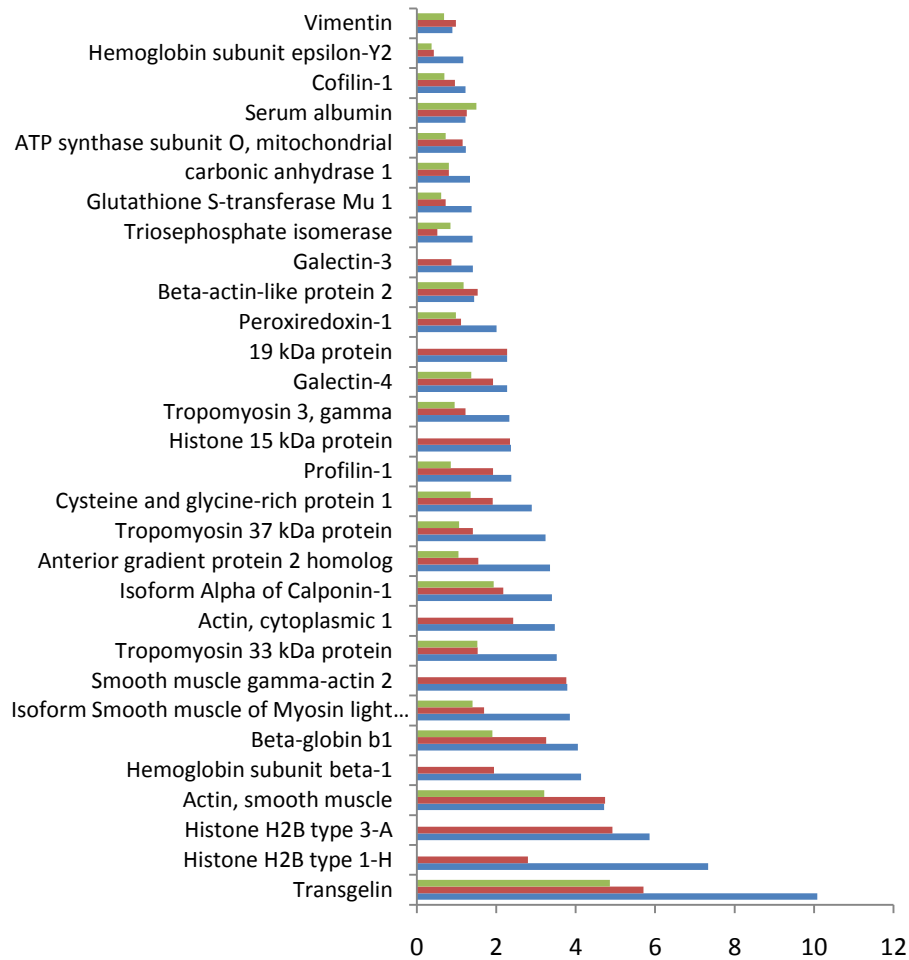

**emPAI**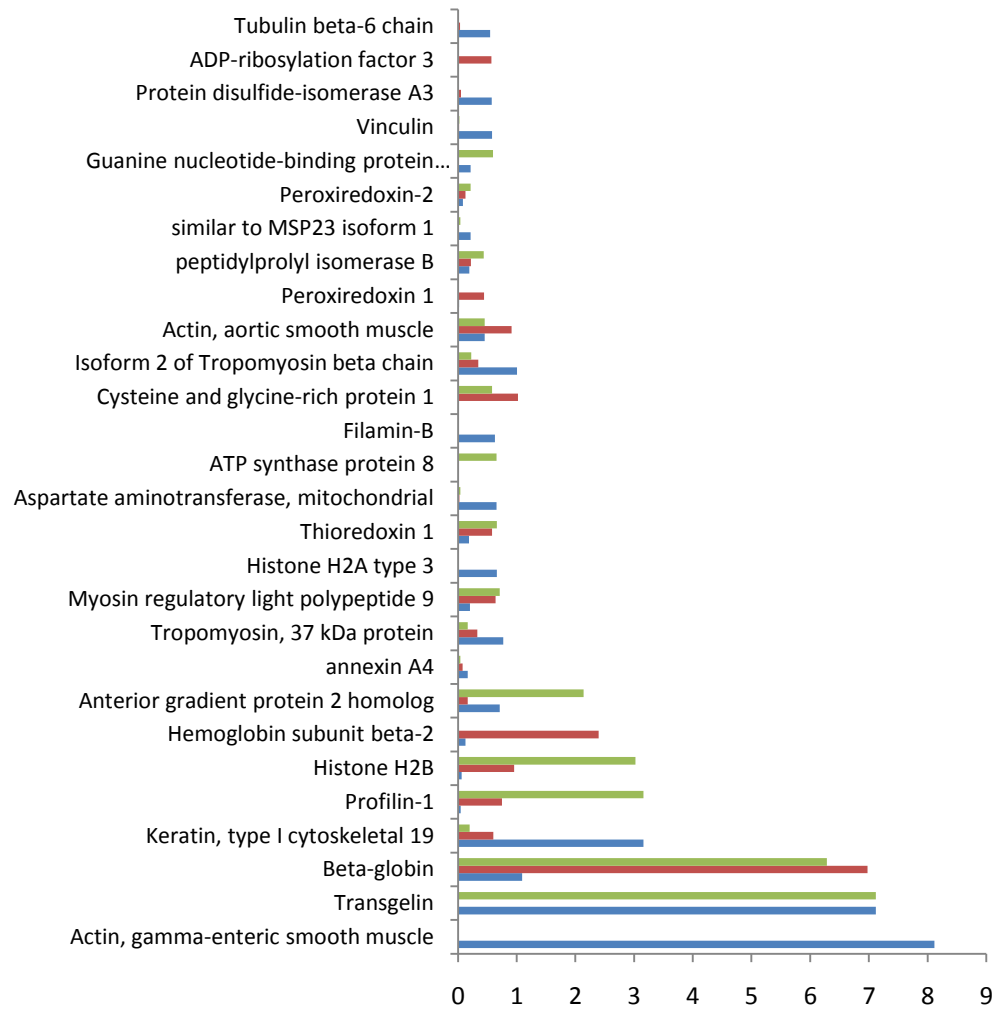

Supplement: Additional file 4 — NSAF {bars representing sample replicate runs}. [file 1756-0381-5-11-S4.pdf]
